# Observations on Diseases in London

**Published:** 1800-04

**Authors:** R. Willan

**Affiliations:** Red Lion Square


### Observations on Diseases in London.

*List of Diseases from the 20th of December to the 15th of March; being the Result of the public and private Practice of a Physician at the West End of the Town.*

| ACUTE DISEASES. | |
|---|---|
| Catarrh | 88 |
| Contagious Malignant Fever | 63 |
| Influenza | 18 |
| Scarlatina Anginosa | 15 |
| Measles | 9 |
| Hooping Cough | 5 |
| Small-Pox | 4 |
| Acute Rheumatism | 20 |
| Rheumatic Pain of the Face | 8 |
| Inflammation of the Lungs | 11 |
| Pleurisy | 2 |
| Inflammation of the Eyes | 4 |
| Inflammatory Sore Throat | 6 |
| Inflammation of the Bowels | 3 |
| Gout | 4 |
| Hæmoptoe | 5 |
| Epistaxis | 2 |
| Intestinal Hæmorrhagy | 6 |
| Renal Hæmorrhagy | 2 |
| Menorrhagia | 8 |
| Abortion | 2 |
| Childbed Fever | 7 |
| Acute Diseases of Infants | 27 |
| Hectic and Slow Fever | 14 |
| Aphthous Fever | 6 |
| Quartan | 1 |
| Tertian | 1 |

| CHRONIC DISEASES. | |
|---|---|
| Cough and Dyspnœa | 151 |
| Pulmonary Consumption | 46 |
| Chronic Rheumatism | 20 |
| Lumbago | 4 |
| Sciatica | 3 |
| Dropsy | 15 |
| Asthenia | 38 |
| Palsy | 6 |
| Spasms of the lower Extremities | 1 |
| Spasms of the Fore Arm | 1 |
| Vertigo and Headach | 11 |
| Epilepsy | 2 |
| Hydrocephalus | 4 |
| Hysteria | 3 |
| Melancholia | 2 |
| Palpitatio | 3 |
| Dyspepsia | 35 |
| Gastrodynia | 19 |
| Enterodynia | 19 |
| Bilious Vomiting | 7 |
| Diarrhœa | 26 |
| Obstipatio and Colic | 6 |
| Chlorosis | 22 |
| Fluor Albus | 5 |
| Scirrhus Uteri | 2 |
| Scirrhus of the Liver | 2 |
| Prolapsus | 2 |
| Gravel and Dysury | 8 |
| Incontinence of Urine | 6 |
| Hæmorrhoids | 3 |
| Jaundice | 14 |
| Worms | 2 |
| Tabes Mesenterica | 7 |
| Scrophula | 8 |
| Bronchocele | 1 |
| Tooth Rash | 3 |
| Lichen | 5 |
| Prurigo | 6 |
| Lepra | 2 |
| Scaly Tetter | 4 |
| Ptyriasis | 3 |
| Erythema | 3 |
| Purpura | 1 |
| Thrush | 4 |
| Herpes | 2 |
| Shingles | 1 |
| Ecthyma | 2 |
| Itch | 5 |
| Impetigo | 4 |
| Porrigo | 7 |
| Lupus | 3 |
| Gutta Rosea | 3 |
| Furunculi | 2 |

Pulmonic diseases were almost universal, and particularly severe in the months of January and February. During the latter month there occurred an epidemic catarrh, chiefly affecting children, and exhibiting the usual symptoms * of the influenza. This disease, although violent for some days, did not in any instance prove fatal. Since the beginning of March, inflammatory and hæmorrhagic complaints have been very prevalent. In the cases of pleurisy and peripneumony, venæsection was employed more than once before the acute pain and sense of constriction about the chest could be relieved.

The short frost in December was not sufficient to put a stop to the progress of the scarlatina anginosa, and typhus or malignant fever, the extent and fatality of which were formerly noticed. During the mild open weather in January, and at the beginning of February, the fever was again rapidly diffused to a very great extent, and with an aggravated train of symptoms. Among the poor, the mortality from this cause was nearly as stated in the last report,† notwithstanding the attentive administration of proper articles of diet, and of suitable remedies, with plenty of wine. The good effects of all these applications are almost wholly superseded by the miserable accommodations of the poor with respect to bedding, and by a total neglect of ventilation in their narrow, crouded dwellings. It will scarcely appear credible, though it is precisely true, that persons of the lowest class do not put clean sheets on their beds three times a year; that even where no sheets are used, they never wash or scour their blankets and coverlets, nor renew them till they are no longer tenable; that curtains, if unfortunately there should be any, are never cleaned, but suffered to continue in the same state till they drop to pieces: lastly, that from three to eight individuals, of different ages, often sleep in the same bed; there being, in general, but one room, and one bed for each family. To the above circumstances may be added, that the room occupied is either a deep cellar, almost inaccessible to the light, and admitting of no change of air; or a garret, with a low roof and small windows, the passage to which is close, kept dark in order to lessen the window tax, and filled not only with bad air, but with putrid, excremental, or other abominable effluvia from a vault at the bottom of the stair-case. Washing of linen, or some other disagreeable business is carried on, while infants are left dozing, and children more advanced kept at play whole days on the tainted

* For an accurate account of these I beg to refer to Medical Communications, Vol. I. page 14.

† i. e. one in four of all persons affected with fever.

tainted bed: ſome unſavoury victuals are from time to time cooked: in many inſtances idleneſs, in others, the cumbrous furniture, or utenſils of trade with which the apartments are clogged, prevent the ſalutary operation of the broom and white-waſhing bruſh, and favours the accumulation of a heterogeneous, fermenting filth.* From all theſe cauſes combined there is neceſſarily produced a complication of fœtor, to deſcribe which would be as vain an attempt, as for thoſe to conceive who have been always accuſtomed to neat and comfortable dwellings.

The above account is not exaggerated: for the truth of it I appeal to the medical practitioners, whoſe ſituation, or humanity has led them to be acquainted with the wretched inhabitants of ſome ſtreets in St. Giles's pariſh, of the courts and alleys adjoining to Liquorpond-ſtreet, Hog Iſland, Turnmill-ſtreet, Saffron-hill, Old-ſtreet, White Croſs-ſtreet, Grub-ſtreet, Golden-lane, the two Brick-lanes, Roſemary-lane, Petticoat-lane, Lower Eaſt Smithfield, ſome parts of Upper Weſtminſter, and ſeveral ſtreets of Southwark, Rotherhithe, &c.

It cannot be wondered at that in ſuch ſituations contagious diſeaſes ſhould be formed, and attain their higheſt degree of virulence. The inhabitants of the ſecond ſtory, in houſes occupied by the poor, are uſually better accommodated; and therefore experience, during ſickneſs of any kind, the beſt effect from public and private charities. But perſons thus ſtationed, ſuffer from contiguity, and from their friendly attentions to thoſe above them, or to the tenants of the cellars; ſo that in whatever part of the houſe a fever commences, it is ſoon diffuſed among all the inmates and their occaſional viſitors, eſpecially in ſeaſons which favour its progreſs, like the laſt autumn and winter. Children, and women conſtantly reſiding in infected apartments, ſeem to get habituated to the action of the fomites. Men and boys, by means of freſh air, and the exerciſe of the day, ſhake off the effects of the virus, and eſcape long unhurt. It muſt, however, be obſerved, that if through taking cold, or any other cauſe, they ſhould be confined to the houſe for ſome days, they aſſuredly take the fever.

* The rooms do not change their condition till they change their tenants: often, indeed, ſo little care is taken, that enough of the old leaven remains to infect all the inmates who ſucceſſively occupy the ſame premiſes. I recollect a houſe in Wood's Cloſe, Clerkenwell, wherein the fomites of fever were thus preſerved for a ſeries of years; at length, a friendly fire effectually cleared away the nuiſance. A houſe, notorious for dirt and infection, near Clare-market, afforded a farther proof of negligence; it was obſtinately tenanted till the wall and floors giving way in the night, cruſhed to death the miſerable inhabitants.

fever. So it happened in the late unfavourable feafon: whoever was obliged to keep his bed for a catarrh, pleurify, or inflammation of the lungs, within three or four days, caught the fever; and almoft every one fo affected died. The children are infected from the new fource of contagion; and the mother, after clofing the eyes of her hufband, and, perhaps, of more than one of her offspring, finks exhaufted with grief, watching, and fatigue, and is, herfelf, the laft victim to the difeafe. The fatality of this fever, within the limits of the Public Difpenfary, has, during the prefent winter, exceeded our former experience, notwithftanding the care of all the medical affiftants.* It is a melancholy confideration that, in London and its vicinity, hundreds, perhaps thoufands of labourers, heads of families, and in the prime of life, are thus configned to perifh annually, being often fo fituated that medical applications, or cordial diet, cannot in any wife alleviate their diftrefs. Perfons in the higher ranks of life are often endangered by the thoughtleffnefs of fervants, who privately vifit their fick friends in infected rooms, and alfo carry thither the children entrufted to their care. Another circumftance, by no means confolatory, is, that linen, and other apparel, fent to laundreffes in clofe parts of the town, muft fometimes return to families, thoroughly impregnated with the effluvia of putrid fever, and of the fcarlatina, &c.

But where is a remedy to be found for fo many evils?—Hofpitals are for the moft part barred againft the entrance of contagious difeafes. Pecuniary aid, whether tranfmitted by the warm heart of benevolence, or wrenched from the flow, reluctant hand of parochial adminiftrators,† is an infufficient palliative for the prefent cafe. Shall the unhappy patient then feek for refuge in the parifh workhoufe? Alas! the fever is already making its ravages there.‡—What therefore is to be done?—All thefe mifchiefs

* My own endeavours were feconded by the abilities and active diligence of my colleague, Dr. Murray; and by the occafional affiftance of Dr. Colladon, Dr. Shea, Dr. T. Smyth, Dr. Baumegarten, Dr. Yelloly, Dr. Ryan, and Dr. W. P. Dimfdale.

† I do not intend this as a general cenfure on parifh officers; many do their duty confciencioufly, but I have been mortified and indignant at the coldnefs with which fome of them receive information of the moft complicated mifery, and at their pofitive refufal to infpect the diftrefs reprefented by the medical attendants.

‡ This has taken place in very many of the workhoufes during the winter; and feveral of the attending furgeons have fuffered feverely from the fever. I am forry to add that our medical friend Dr. Yelloly was confined three weeks by a malignant fever taken near Brook's Market from a family, all the individuals of which were previoufly relieved by his care, and the co-operation of the overfeers.

chiefs admit of ready alleviation, and might, with proper management, be removed at a moderate expence. Let Houfes of Recovery be eftablifhed in open, airy fituations, at fome diftance from other buildings, but adjoining to different diftricts of the metropolis; to be fupported either at the joint expence of the feveral parifhes within each diftrict, or by a voluntary fubfcription among its principal inhabitants. As foon as any perfon exhibits fymptoms of a fever from infection let him be inftantly removed into the Houfe of Recovery, where, being wafhed, and put with clean linen into a frefh bed, he will foon be freed from his complaints, and able to rejoin his wife and family. To them, in the mean time, a loan of bedding fhould be made till their own bed is cleanfed, and till the walls and floor are wafhed, or fcoured. The revenue neceffary to fupport houfes inftituted on fuch a plan is not fo great as might be imagined. Both the utility and expence of them have been already put to a noble trial by the merchants, and manufacturers of the populous town of Manchefter. Their example deferves to be followed in this metropolis, and all other great cities; the neceffity of a receptacle for contagious fevers being always proportioned to the magnitude of the place. The fame receptacles might occafionally ferve for the relief of afthmatic, confumptive, and other pulmonic difeafes, which predominate, or are aggravated at a feafon when fevers are nearly extinct. One-fourth, and in very unfavourable feafons one-third of all the deaths in London, is, according to the bills of mortality,* caufed by difeafes of the lungs; a circumftance which furely merits fome confideration. Thefe complaints are univerfally, and perhaps with reafon, excluded from hofpitals: they require a free circulation of pure air, and admit of little relief where patients are confined to fmall rooms in clofe narrow courts and alleys. Pulmonic difeafes, however, although fo fatal in themfelves, extend no farther than the individuals affected with them; whereas the fcarlet fever, fimple typhus, and typhus anginofus or malignant fore throat, through the medium of infectious fomites, endanger the health and peace of the whole community. Such difeafes, therefore, fhould be the more immediate object of attention: and I thought it my duty, after obferving fo great a mortality, to found an alarm to our fellow citizens; to

* From the London bills of mortality it appears, that in the year 1796 there died of pulmonic diforders 5,910 out of 18,238; in the year 1797 of pulmonic diforders 5,439 out of 16,714; and in the year 1799 of pulmonic diforders 6,210 out of 17,285. The articles of abortive and ftill-born, of violent deaths, and cafualties, are neceffarily excluded from the lift. See the Account of Difeafes in London, Monthly Magazine for April, 1797.

to state the origin, causes, and rapid diffusion, at some seasons, of putrid, infectious diseases; and to point out the means of preventing the calamities, and devastation annually caused in an useful class of people, and extended from them to the superior ranks.

Having given my sentiments, and the result of my own knowledge on the subject, I cannot but do justice to those who first carried into execution the plan above recommended, and to others, who, probably before me, have thought it applicable to the state of the poor in London. It is, therefore, with satisfaction, I refer to Dr. Ferriar's Medical Essays; and to a transcript from them, published in the last report of the Society for bettering the condition of the poor, by the unwearied philanthropist Thomas Bernard, Esq. treasurer of the Foundling Hospital. I am happy to observe farther, that the treasurer of the Public Dispensary, William Waddington, Esq. (Sloane Street) has been struck with the necessity of houses for the reception of infectious diseases, and is ready to co-operate in their establishment near the metropolis. I need not add, what has I believe been fully impressed on the public mind from his various exertions in the cause of humanity, that this gentleman is not in the habit of leaving unfinished what he once undertakes.

*Red Lion Square, March* 30, 1800. R. WILLAN.

*** On receiving the above report, Dr. Bradley informs me that the medical gentlemen connected with the Westminster Hospital formerly held some conversations on the same subject; but that no measures were taken for carrying the design into execution.

---

